# Mechanisms of viral mutation

**DOI:** 10.1007/s00018-016-2299-6

**Published:** 2016-07-08

**Authors:** Rafael Sanjuán, Pilar Domingo-Calap

**Affiliations:** 1 Department of Genetics and Institute for Integrative Systems Biology (I2SysBio), Universitat de València, C/Catedrático José Beltrán 2, 46980 Paterna, Valencia Spain; 2 Laboratoire d’ImmunoRhumatologie Moléculaire, INSERM UMR_S1109, LabEx Transplantex, Centre de Recherche d’Immunologie et d’Hématologie, Faculté de Médecine, Fédération de Médecine Translationnelle de Strasbourg (FMTS), Université de Strasbourg, Strasbourg, France; 3 Fédération Hospitalo-Universitaire OMICARE, Centre de Recherche d’Immunologie et d’Hématologie, Strasbourg, France

**Keywords:** Virus, Mutation rate, Replication fidelity, Hyper-mutation, Post-replicative repair, Genetic diversity, Evolution

## Abstract

The remarkable capacity of some viruses to adapt to new hosts and environments is highly dependent on their ability to generate de novo diversity in a short period of time. Rates of spontaneous mutation vary amply among viruses. RNA viruses mutate faster than DNA viruses, single-stranded viruses mutate faster than double-strand virus, and genome size appears to correlate negatively with mutation rate. Viral mutation rates are modulated at different levels, including polymerase fidelity, sequence context, template secondary structure, cellular microenvironment, replication mechanisms, proofreading, and access to post-replicative repair. Additionally, massive numbers of mutations can be introduced by some virus-encoded diversity-generating elements, as well as by host-encoded cytidine/adenine deaminases. Our current knowledge of viral mutation rates indicates that viral genetic diversity is determined by multiple virus- and host-dependent processes, and that viral mutation rates can evolve in response to specific selective pressures.

## Introduction

**Fig. 1:**
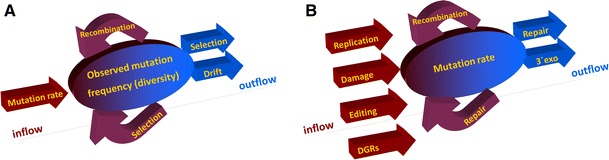
Mutation rate definition. **a** Basic processes determining population genetic diversity. The observed population frequency of a mutation depends on the rate at which it is produced (mutation rate), but also on natural selection, random genetic drift, and recombination, among other processes. Most mutations are deleterious and tend to be removed from the population by selection, whereas beneficial mutations or combinations of mutations can be maintained/favored. Recombination can also contribute to the maintenance of genetic diversity. Random genetic drift leads to allele fixation and hence reduces population genetic diversity. **b** Basic processes determining viral mutation rates. Mutations originate from replication errors, nucleic acid damage, and editing of the genetic material by host-encoded proteins or by specialized molecular systems such as diversity-generating retro-elements (DGRs). If these changes are not corrected, they will be passed to the viral progeny and hence will contribute to elevating the viral mutation rate. Expression of host error-prone polymerases may also contribute to creating new mutations in viruses. Recombination can also enhance the ability of some viruses to create new mutations by increasing gene copy number or by producing genome rearrangements

The mutation rate of an organism is defined as the probability that a change in genetic information is passed to the next generation. In viruses, a generation is often defined as a cell infection cycle, which includes attachment to the cell surface, entry, gene expression, replication, encapsidation, and release of infectious particles. Mutations are not restricted to replication since they can also result from editing of the genetic material, or spontaneous nucleic acid damage. The mutation rate should not be confused with the frequency at which mutations are found in a given viral population. The latter is a measure of genetic variation that depends on a number of other processes such as natural selection, random genetic drift, recombination, and so on (Fig. 1a). Higher mutation rates lead to higher genetic diversity but, except in special cases, it is not possible to infer mutation rates directly from observed population mutation frequencies [1]. Although genetic diversity depends on multiple factors, the mutation rate is of particular interest because it constitutes the ultimate source of genetic variation. Similarly, mutation rates should not be confused with molecular evolutionary rates. The neutral theory of molecular evolution posits a linear relationship between these two rates, but whereas mutation is a biochemical/genetic process, molecular evolution refers to the fixation of new alleles in populations [2, 3].

Knowledge of the processes underlying viral mutation rates has implications for understanding and managing drug resistance, immune escape, vaccination, pathogenesis, and the emergence of new diseases. In clinics, the importance of viral mutation rates can be illustrated by the history of anti-HIV treatment. The nucleoside analog azidothymidine (AZT) was the first approved anti-HIV drug but, unfortunately, the appearance of drug-resistant variants rapidly frustrated this monotherapy. HIV-1 is a fast-mutating virus and produces every possible single-base substitution (including AZT-resistance mutations) within a patient everyday [4]. The subsequent success of highly active antiretroviral therapy did not reside on merely increasing drug potency but mainly in combining different drugs (including AZT), such that the chances of resistance mutations appear were minimized. Qualitatively, the same argument holds for other rapidly mutating viruses such as hepatitis C virus (HCV). Multiple resistances have been already described against new HCV treatments [5], and analysis of population sequences has shown that resistance to protease inhibitors and non-nucleoside polymerase inhibitors pre-exist naturally in treatment-naïve patients, that is, in the absence of selection favoring these mutations [6]. At present, combination therapies are the only effective treatment strategy for chronic diseases caused by fast-mutating viruses.

A similar scenario can be depicted for antiviral immunity. Viruses showing high mutation rates tend to evade immunity more efficiently. There are numerous examples of cytotoxic T lymphocyte (CTL) and antibody evasion in HIV-1, HCV, and hepatitis B virus (HBV), three fast-mutating viruses causing chronic infections. In HBV, the most common cause of hepatitis worldwide with nearly 350 million people chronically infected, a series of point mutations have been associated with immune escape and vaccination failure [7]. In acute viruses, immune escape takes place at the host population level instead of at the intra-host level. In this case, the benefit of escape resides in the ability of the virus to re-infect hosts that have developed protective immunity or infect hosts with that recognize the same antigens. The best-known example is influenza virus, which constantly undergoes antigenic changes and therefore requires yearly vaccine updates. Current efforts focus on developing influenza vaccines that target evolutionarily more conserved, yet sufficiently immunogenic protein domains [8]. Viral genetic diversity, which is ultimately determined by mutation rates, has therefore a profound effect on the design of antiviral strategies.

Viral mutation rates are not merely caused by polymerase errors, but also by the ability of a virus to correct DNA mismatches by proofreading and/or post-replicative repair. Furthermore, other sources of mutation include host enzymes, spontaneous nucleic acid damage, and even special genetic elements located within some viral genomes whose specific function is to produce new mutations (Fig. 1b). Mutation rates are modulated by additional factors, including proteins involved in replication other than the polymerase, the mode of replication, and the template sequence and structure. In this review, we discuss how these different factors control viral mutation rates.

## RNA viruses versus DNA viruses

**Fig. 2:**
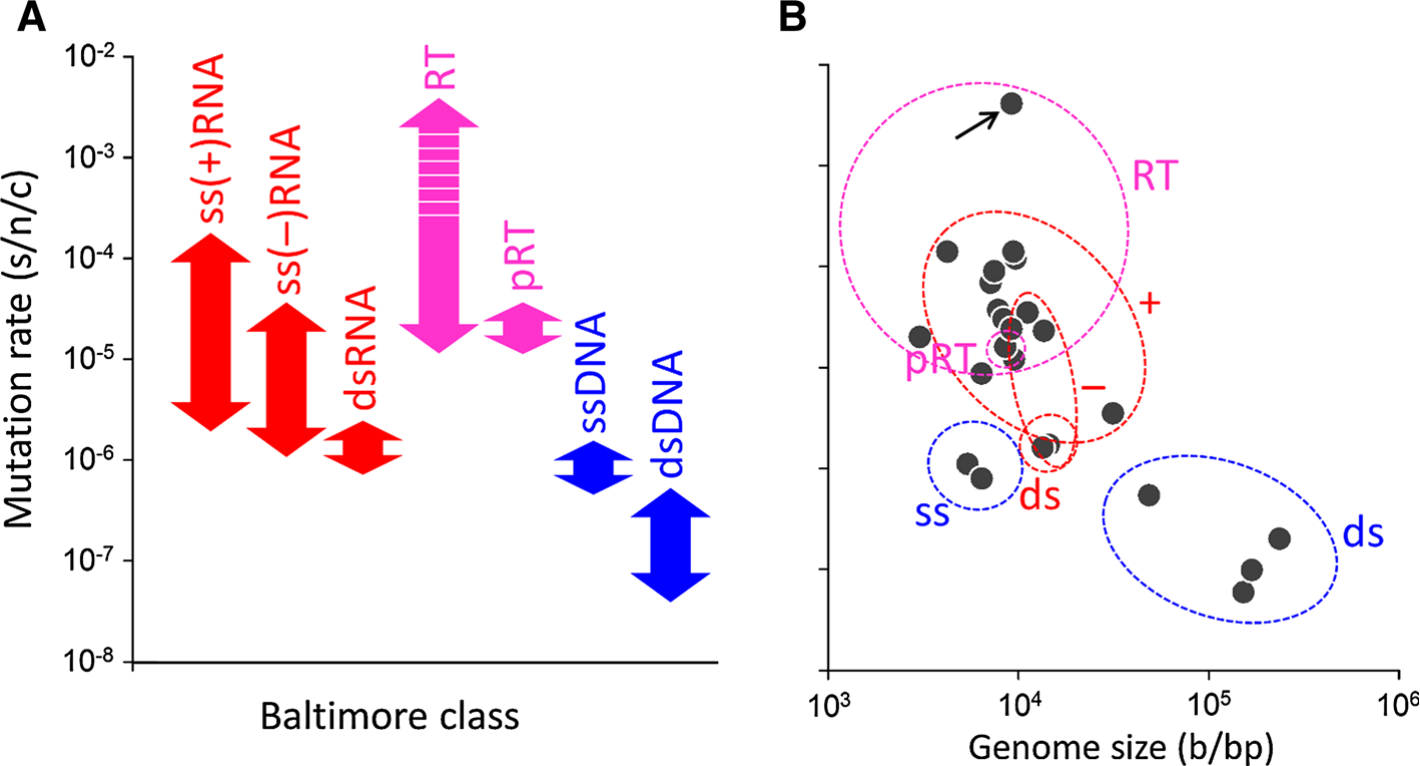
Mutation rate variation across viruses. **a** Range of variation of mutation rates for the seven Baltimore classes of viruses (*ss* single-strand, *ds* double-strand; +/− genome polarity, *RT* retroviruses, *pRT* para-retroviruses). In the RT group, all mutation rates fall in the *non-hatched* *arrow* region except the HIV-1 mutation rate measured in cellular DNA, which is orders of magnitude higher than the rate measured in plasma. This is because many APOBEC-edited viral genomes fail to produce viable progeny and hence do not reach plasma (see text for details). **b** Negative correlation between genome size and mutation rate in viruses. Baltimore groups are indicated. The observed correlation can be explained in terms of differences between RNA and DNA viruses and between ss and ds viruses. In the RT group, the extremely high mutation rate of HIV-1 in cellular DNA is indicated with an *arrow*. In contrast, the HIV-1 mutation rate measured in plasma falls within the usual RT range

The Baltimore classification of viruses establishes the following categories according to the genetic material contained in the virion: positive-strand RNA viruses (e.g., rhinoviruses, hepatitis C virus, noroviruses, tobacco mosaic virus), negative-strand RNA viruses (influenza viruses, Ebola virus, rabies virus), double-strand RNA viruses (rotaviruses, bursal disease virus), retroviruses (HIV, human T cell leukemia virus), para-retroviruses (hepatitis B viruses), single-stranded DNA viruses (parvoviruses, bacteriophage ϕX174), and double-stranded DNA viruses (papillomaviruses, herpesviruses, adenoviruses, poxviruses). Viruses are the biological systems with the widest variation in mutation rates, the largest differences being found between RNA and DNA viruses. A summary of mutation rates for different viruses is provided in Table 1. As discussed in previous work, the reliability of some of these rates is compromised by several sources of estimation error and bias [1]. Despite these uncertainties, it can be inferred that viral mutation rates roughly range between 10^−8^ and 10^−4^ substitutions per nucleotide per cell infection (s/n/c), with DNA viruses occupying the 10^−8^–10^−6^ range and RNA viruses the 10^−6^–10^−4^ range (Fig. 2a). These differences have several mechanistic bases. First, the polymerases of the vast majority of RNA viruses lack 3′ exonuclease proofreading activity and hence are more error-prone than those of DNA viruses [9, 10]. The exception to this rule is provided by coronaviruses, a family of positive-strand RNA viruses encoding a complex RNA-dependent RNA polymerase that has a 3′ exonuclease domain [11]. Reverse transcriptases (RTs) also lack 3′ exonuclease activity [12, 13] and, hence, retroviruses (viruses with RNA-containing virions and a cellular DNA stage) and para-retroviruses (viruses with DNA-containing virions and a cellular RNA stage) mutate and evolve at rates similar to those of non-reverse transcribing RNA viruses (the latter are often called riboviruses).

**Table 1:** Summary of viral mutation rates

Class	Virus	Genome size (kb)	Average mutation rate (s/n/c)^a^	Individual estimates (s/n/c)^b^ and references
ss(+)RNA	Bacteriophage Qβ^c^	4.22	1.4 × 10^−4^	1.4 × 10^−4^ [29]
	Tobacco mosaic virus	6.40	8.7 × 10^−6^	8.7 × 10^−6^ [128]
	Human rhinovirus 14	7.13	6.9 × 10^−5^	4.8 × 10^−4^ [129], 1.0 × 10^−5^ [130]
	Poliovirus 1	7.44	9.0 × 10^−5^	2.2 × 10^−5^ [131, 132], 1.1 × 10^−4^ [133], 3.0 × 10^−4^ [134]
	Human norovirus G1	7.65	1.5 × 10^−4^	1.5 × 10^−4^ [74]
	Tobacco etch virus	9.49	1.2 × 10^−5^	3.0 × 10^−5^ [135], 4.8 × 10^−6^ [136]
	Hepatitis C virus	9.65	3.8 × 10^−5^	1.2 × 10^−4^ [137], 2.5 × 10^−5^ [138], 2.0 × 10^−5^ [138], 3.5 × 10^−5^ [105]
	Murine hepatitis virus	31.4	3.5 × 10^−6^	3.5 × 10^−6^ [139]
ss(−)RNA	Vesicular stomatitis virus	11.2	3.7 × 10^−5^	6.9 × 10^−5^ [140, 141], 1.8 × 10^−5^ [142], 4.2 × 10^−5^ [143]
	Influenza A virus	13.6	2.5 × 10^−5^	4.5 × 10^−5^ [144], 7.1 × 10^−6^ [145], 3.9 × 10^−5^ [146], 3.1 × 10^−5^ [147]
	Measles virus^d^	15.9	3.5 × 10^−5^	2.8 × 10^−5^ [148], 4.4 × 10^−5^ [149]
dsRNA	Bacteriophage Φ6	13.4	1.6 × 10^−6^	1.6 × 10^−6^ [82]
Reverse transcribing	Duck hepatitis B virus	3.03	2.0 × 10^−5^	2.0 × 10^−5^ [150]
	Spleen necrosis virus	7.80	3.7 × 10^−5^	2.4 × 10^−5^ [151], 5.8 × 10^−5^ [152]
	Murine leukemia virus	8.33	3.0 × 10^−5^	6.0 × 10^−6^ [153], 4.2 × 10^−5^ [154], 1.1 × 10^−4^ [155, 156]
	Bovine leukemia virus	8.42	1.7 × 10^−5^	1.7 × 10^−5^ [157]
	Human T-cell leukemia virus	8.50	1.6 × 10^−5^	1.6 × 10^−5^ [158]
	HIV-1 (free virions)	9.18	6.3 × 10^−5^	4.9 × 10^−5^ [76, 159, 160], 1.0 × 10^−4^ [161], 8.7 × 10^−5^ [162], 4.4 × 10^−5^ [163], 3.6 × 10^−5^ [99], 9.3 × 10^−5^ [63]
	HIV-1 (cellular DNA)	9.18	4.4 × 10^−3^	4.4 × 10^−3^ [63]
	Foamy virus	13.2	2.1 × 10^−5^	2.1 × 10^−5^ [164]
	Rous sarcoma virus	9.40	1.4 × 10^−4^	1.4 × 10^−4^ [165]
ssDNA	Bacteriophage ΦX174	5.39	1.1 × 10^−6^	1.3 × 10^−6^ [166], 1.0 × 10^−6^ [23]
	Bacteriophage m13	6.41	7.9 × 10^−7^	7.9 × 10^−7^ [94]
dsDNA	Bacteriophage λ	48.5	5.4 × 10^−7^	5.4 × 10^−7^ [167, 168]
	Herpes simplex virus	152	5.9 × 10^−8^	5.9 × 10^−8^ [169, 170]
	Bacteriophage T2	169	9.8 × 10^−8^	9.8 × 10^−8^ [167, 171]
	Human cytomegalovirus	235	2.0 × 10^−7^	2.0 × 10^−7^ [20]

^a^Geometric mean of the individual estimates

^b^Mutation rates were normalized to s/n/c units as detailed in previous work [1]

^c^This corresponds to a consensus estimate from several studies, see original publication for details

^d^Assuming linear replication, see original references for details

Whereas the dichotomy between RNA/RT and DNA viruses is well established from genetic and mechanistic standpoints, differences are less clear from the point of view of molecular evolution [14]. Some DNA viruses have been shown to evolve at rates close to those of RNA viruses, including emerging canine parvovirus strains [15], human parvovirus [16], tomato yellow leaf curl geminivirus [17], beak-and-feather disease circovirus [18], and African swine fever virus (ASFV) [19], among others. This underscores the fact that evolution depends on multiple factors other than mutation rate, but also that mutation rates are unknown for many DNA viruses and may, in some cases, be higher than currently believed. Recent work with human cytomegalovirus has suggested a genome-wide average of 2 × 10^−7^ s/n/c, a value slightly higher than previously thought for a large double-strand DNA virus [20], although this estimate was indirect. Since many DNA and RNA viruses share similar lifestyles, the question arises as to why mutation rates should have evolved so differently in these two broad groups.

## Single-strand viruses show higher mutation rates than double-strand viruses

Single-strand DNA viruses tend to mutate faster than double-strand DNA viruses, although this difference is based on work with bacteriophages, as no mutation rate estimates have been obtained for eukaryotic single-strand DNA viruses [1]. Within RNA viruses, there are no obvious differences in mutation rate among Baltimore classes (Fig. 2a). The mechanisms underlying these differences are not well understood. One possible explanation for the differences between single and double-strand viruses is that single-strand nucleic acids are more prone to oxidative deamination and other types of chemical damage. Elevated levels of reactive oxygen species (ROS) and other cellular metabolites during viral infections can induce mutations in the host cell and in the virus. For instance, ethanol is likely to synergize with virus-induced oxidative stress to increase the mutation rate of HCV [21]. Differences among single- and double-strand DNA viruses may also be explained in terms of their access to post-replicative repair. Work with bacteriophage ϕX174 has provided interesting clues on this issue. In enterobacteria, methyl-directed mismatch repair (MMR) is performed by MutHLS proteins and Dam methylase. Dam methylation of GATC sequence motifs is used to differentiate the template and daughter DNA strands and is thus required to perform mismatch correction [22]. Mismatches are recognized by MutS, which interacts with MutL and leads to the activation of the MutH endonuclease, which excises the daughter strand. However, the genome of bacteriophage ϕX174 has no GATC sequence motifs, even if approximately 20 such sites are expected by chance. As a result, the ϕX174 DNA cannot undergo MMR. This contributes to explaining the relatively high mutation rate of this virus, which falls on the order of 10^−6^ s/n/c, a value three orders of magnitude above that of *Escherichia coli* and highest among DNA viruses [23]. Avoidance of GATC motifs may be a consequence of selection acting on mutation rate, but also of other selective factors. For instance, inefficient methylation of the phage DNA may render it susceptible to cleavage by MutH, therefore imposing a selection pressure against GATC sequence motifs [24].

As opposed to bacteriophage ϕX174, the link between post-replicative repair and mutation rate is still unclear in eukaryotic viruses. Numerous studies have shown that viruses interact with DNA damage response (DDR) pathways by altering the localization or promoting the degradation of DDR components [25, 26]. For instance, the adenoviral E4orf6 protein promotes proteasomal degradation of TOPBP1, a DDR component [27]. DDR activation can occur as an indirect consequence of cellular stress due to the infection per se or as a part of an antiviral response, which would be in turn counteracted by viruses. Although DNA viruses tend to promote genomic instability in the host cell, it remains to be shown whether DDR dysregulation can determine DNA virus mutation rates.

## Viruses with smaller genomes tend to mutate faster

A general inverse correlation between genome size and mutation rate applies to DNA-based microorganisms including viruses, bacteria and unicellular eukaryotes [28]. According to this rule, the per-genome mutation rate stays relatively constant at a value of approximately 0.003 per round of copy. A similar negative relationship seems to exist in RNA viruses, but their smaller genome size range of variation makes it more difficult to detect such trend (Fig. 2b). Supporting this correlation, however, coronaviruses have the largest genomes among RNA viruses (30–33 kb) and have evolved proofreading capacity, as opposed to all other RNA viruses known [11]. Conversely, one of the highest mutation rate described for a ribovirus corresponds to bacteriophage Qβ, which has one of the smallest RNA genomes [29]. Therefore, there appears to be a general negative correlation between mutation rates and genome size in microorganisms. However, the underlying causes remain unclear, both at the mechanistic and evolutionary levels. First, there are no known differences in intrinsic replication fidelity among the polymerases of different RNA viruses (excepting coronavirus exonuclease activity). Second, in DNA viruses, those with higher estimated mutation rates have smaller genomes, but also have single-strand DNA (Fig. 2). Estimates for small double-strand DNA viruses would be needed to clarify which of these two factors contributes more to elevating mutation rates. The observation that most highly variable and rapidly evolving DNA viruses have small genomes (including double-strand viruses) indirectly supports an effect of genome size [3].

Candidate mechanisms that might account for mutation rate differences between large and small DNA viruses may involve virus–DDR interactions. Whereas many viruses appear to evade DDR, others seem to use it for their own benefit [25, 26]. Polyomaviruses, papillomaviruses and parvoviruses induce and depend on DDR signaling pathways for efficient replication [30–32]. These viruses share the property of having small, circular DNA genomes which do not encode a polymerase. As such, they depend directly on the cellular replication machinery, as opposed to larger DNA viruses. It is possible that some small viruses promote the DDR to prolong the S cell-cycle phase, which offers a more favorable environment for replication. By adopting circular genomes, these viruses would also avoid the formation of genome concatemers, a typical effect of DDR in linear viral genomes such as, for instance, adenoviruses [33]. Whether differences in DDR activation between small/circular and large/linear DNA viruses translate into mutation rate differences remains to be tested. The DDR comprises error-prone DNA polymerases for re-synthesis of excised strands [34], and involvement of these polymerases in viral replication may lead to higher mutation rates.

## Polymerase fidelity variants

Intrinsic polymerase fidelity (i.e., the ability to incorporate the correct base and exclude incorrect bases from the active site during DNA synthesis) is a primary mutation rate determinant. Polymerase variants with altered fidelity have been artificially selected in a number of RNA viruses by subjecting laboratory populations to mutagenic treatments [35]. For instance, serial passaging of poliovirus in the presence of the base analog ribavirin led to the selection of a polymerase variant (G64S) with threefold increased fidelity [36]. This same mutation also confers increased fidelity in the related human enterovirus 71 [37], and other amino acid replacements such as L123F have also been shown to modify the replication fidelity of this virus [38]. Passaging of coxsackievirus B3 (also a member of the enterovirus genus in the picornavirus family) in the presence of ribavirin or 5-azacytidine selected for another fidelity variant in the viral polymerase (A372V) [39]. Outside picornaviruses, fidelity variants have been more recently obtained by serial mutagen treatment in chikungunya virus [40], influenza A virus [41], and West Nile virus [42]. Several antivirals and notably many antiretroviral drugs are base analogs. Resistance to these treatments is well documented in the HIV-1 RT and some of these variants modify replication fidelity, as determined in vitro or in cell cultures [13]. Intrinsic fidelity can be determined by residues located inside or outside the catalytic domain [43, 44]. For instance, reorientation of the triphosphate moiety of the incoming nucleotide is a fidelity checkpoint in poliovirus polymerase [45]. Interestingly, recent work has shown that replication fidelity can also be determined by proteins of the replication complex other than the viral polymerase. Serial passages of chikungunya virus in the presence of nucleoside analogs favored the appearance of substitution G641D in the RNA helicase nsP2 [40]. This variant increased replication fidelity through mechanisms linked to reduced helicase activity, increased replication kinetics, and resistance to low nucleotide concentrations [46]. Fidelity variants demonstrate the ability of RNA viruses to evolutionarily adjust mutation rates in response to selection acting on mutation rate or other traits.

DNA virus mutation rates also respond to selection, as shown in earlier work with bacteriophage T4 in which a series of polymerase variants were identified following chemical mutagenesis [47]. T4 polymerase variants showing strongly increased fidelity have been described (as opposed to more modest effects in RNA viruses) and tend to map to the central palm and the carboxyl-terminal thumb subdomain of the viral polymerase. Mutator phenotypes have also been described in T4. This phenotype can be conferred by changes in replication factors such as single stranded DNA-binding proteins or helicase proteins [48]. However, the strongest mutator phenotypes (up to 400-fold increase in mutation rate) often result from 3′ exonuclease inactivation in T4 [47]. Similar results were obtained with herpes simplex virus type 1 (HSV-1), for which mutations in the conserved regions of the polymerase domain were found to modify replication fidelity. A HSV-1 polymerase mutant containing Y577H/D581A substitutions was exonuclease-deficient and exhibited a mutator phenotype. However, this variant rapidly evolved a compensatory substitution (L774F) that restored DNA replication fidelity in this genetic background [49, 50]. Since RNA virus polymerases typically lack this activity, no such mutators can be produced, except for coronaviruses [51]. Furthermore, the genetic diversity of RNA viruses is probably closer to an upper tolerability limit beyond which the population genetic load increases to levels incompatible with virus survival [3, 52]. Therefore, both biochemical and population-genetic factors limit the appearance of strong mutators in RNA viruses.

## Host-encoded mutation rate modifiers in RNA and reverse-transcribing viruses

Whereas post-replicative repair probably plays a role in determining DNA virus mutation rates (as discussed above), RNA virus mutation rates are strongly influenced by other host-encoded factors. Apolipoprotein B mRNA-editing catalytic polypeptide-like enzymes (APOBEC) are a family of cellular cytidine deaminases that function as an innate cellular defense against retroviruses [53]. This family has expanded and diverged throughout vertebrate evolution and includes five APOBECs [54]. APOBEC3G was first shown to massively convert cytidines to uracils in the complementary HIV-1 DNA during or following reverse transcription [55–57]. APOBEC activity is antagonized by the viral protein Vif, which binds to and promotes the proteasomal degradation of APOBEC [58]. There are seven APOBEC3 paralogs in the human genome (A–D and F–H) which have been shown to also edit retroelements and other viruses, including hepatitis B virus [59], papillomaviruses [60], and herpesviruses [61]. Editing is strongly dependent on sequence-context. The major determinant of editing for human APOBECs is the −1 base, thus defining typical dinucleotide targets (the edited base and the −1 base). APOBEC3G prefers CC dinucleotides whereas the other APOBEC forms prefer TC dinucleotides. DNA editing hotspots have been identified and depend both on sequence context and DNA secondary structure [62]. In HIV-1, editing of the complementary DNA strand produces GG-to-AG or GA-to-AA mutations in the genomic RNA. In recent work, we have estimated the relative contributions of host APOBECs and the viral RT to the total HIV-1 mutation rate in vivo [63]. We found that the vast majority of mutations (98 %) are produced by APOBECs and that this elevates the HIV-1 mutation rate by >40-fold above the RT error rate, making HIV-1 the fastest mutating virus described so far. In many cases, hyper-mutation leads to loss of infectivity and hence effectively exerts its antiviral action. However, APOBECs can also produce moderately mutated, viable viruses, thus raising the question whether these deaminases may contribute to viral diversity and evolution, immune escape, and drug resistance [64–66].

Double-strand RNA-dependent adenosine deaminases (ADARs) are another type of host enzymes that edit viral genomes by deaminating adenosines in long double-stranded RNA and converting them to inosines. The latter base-pair with guanosines, resulting in A-to-G base substitutions [67]. ADARs also exhibit sequence context preferences, although less marked than in the case of APOBECs [68]. ADAR-driven hyper-mutation was first demonstrated in measles virus [69] and has since been suggested for a variety of RNA viruses including human parainfluenza virus [70], respiratory syncytial virus [71], lymphocytic choriomeningitis virus [72], Rift Valley fever virus [73], and noroviruses [74].

Lastly, other cellular proteins such as uracil DNA glycosylases (UNG) can modulate viral mutation rates. Uracil can be found in DNA abnormally due to spontaneous or enzymatically induced cytidine deamination, leading to G-to-A mutations. To avoid the deleterious effects of uracil in DNA, UNG recognizes and excises uracil residues present in DNA. The HIV-1 protein Vpr interacts with UNG and mediates its incorporation into HIV-1 virions. Failure to incorporate UNG produces a fourfold increase of the HIV-1 mutation rate in actively dividing cells, and of 18-fold in macrophages [75, 76]. Variations in the concentration and balance of dNTPs among cell types may also influence viral mutation rates [77]. Although analysis of HIV-1 mutations in various cell lines revealed no obvious mutation rate differences, it nevertheless showed differences in the type of mutations produced [78].

## Mutation accumulation is determined by replication mode

**Fig. 3:**
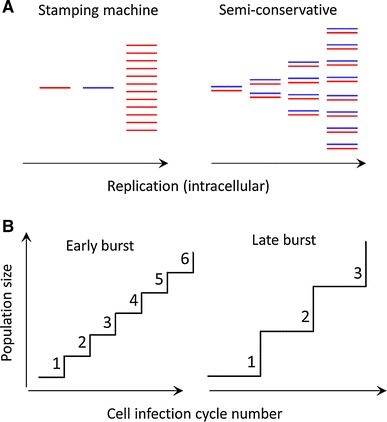
Viral replication modes and mutation accumulation. **a** Stamping machine versus semi-conservative replication. As opposed to cells, which use only semi-conservative replication, viruses can adopt a variety of replication modes. In the stamping machine model, a single template strand is used to synthesize all progeny genomes within a given cell. However, this is not possible in practice because replication requires synthesis of complementary strands or “anti-genomes” (*blue*). Under this model, the mutation frequency after one cell infection cycle will equal the mutation rate except if mutations occur during the first round of copying (from genome to anti-genome), in which case they will be present in all of the viral progeny. Under semi-conservative replication, multiple rounds of copying are required to produce enough progeny, thus allowing for the intra-cellular accumulation of mutations. **b** Relationship between lysis time and mutation accumulation. Longer cell infection cycles (late burst) can allow for the production of more progeny viruses. Under semi-conservative replication, this will require more rounds of copying but, if replication follows the stamping machine model, the number of rounds of copying will not change (more progeny genomes will be produced from the same template). Hence under this model, a late-burst virus variant will undergo fewer total rounds of copying at the population scale than early-burst variants and will tend to accumulate fewer mutations

In contrast to cells, viruses can adopt a variety of replication modes. Replication is said to follow a “stamping machine” model if a single template is used to produce all progeny strands within a given cell (Fig. 3a). Under this theoretical model, there is only one round of copying per cell. In practice, this means that each infecting genome is used to synthesize a single reverse-complementary intermediate which in turn is used as template for synthesizing all progeny genomes. This contrasts with semi-conservative replication, in which each strand is copied once to produce progeny molecules that are, in turn, used as templates in the next round of copying. Since under semi-conservative replication the number of strands doubles in each cycle, the virus necessarily has to undergo multiple replication cycles within each cell to produce enough progeny. Under stamping machine replication the mutation frequency observed after one cell infection equals the mutation rate, but under semi-conservative replication this frequency is also determined by the number of replication cycles, as mutants become amplified. This means that a given viral polymerase will produce more mutations per cell if replication is semi-conservative than if replication is stamping machine-like. These two models are indeed two extremes of a continuum of possible replication modes. For instance, a virus can produce multiple progeny molecules per round of copying which then undergo a second replication cycle in the same cell to end up producing hundreds or thousands of progeny molecules.

It has been suggested that the stamping machine model has been selectively favored in RNA viruses because it compensates for the extremely high error rate of their polymerases [79–81]. Some RNA viruses such as bacteriophage ϕ6 [82], bacteriophage Qβ [83] and turnip mosaic virus [84] tend to replicate via the stamping machine model. However, empirically-informed modeling of the poliovirus replication cycle indicated multiple rounds of copying per cell [85]. Similarly, single-cell analysis of the genetic diversity produced by vesicular stomatitis virus revealed that some mutations are amplified within cells, implying that multiple rounds of copying take place per cell [86]. However, it remains unknown whether a given virus can modify its replication mode in response to specific selective pressures in order to promote or down-regulate mutational output. To a large extent, the replication mode of most viruses should be dictated by the molecular mechanisms of replication and, hence, should be subjected to strong functional constraints. For instance, bacteriophage ϕX174 replicates via the stamping machine mode because it uses rolling circle replication [87, 88]. In contrast, semi-conservative replication is probably the only mechanistically feasible replication model for viruses with large DNA genomes.

## Lysis time as a regulator of mutational output

Changes in lysis time can be thought of as another mechanism for regulating the production of mutations in viral populations. Lysis is a tightly regulated process and, in theory, viral fitness is maximized for some intermediate lysis time [89–91]. If lysis occurs before this optimum, the infected cell will release a small amount viral progeny and hence few cells will be infected in the next infection cycle, retarding population growth. Yet if lysis occurs after the optimum, a large amount of progeny will be produced per cell but cell-to-cell transmission will be delayed. The optimal lysis time depends on the time required to start producing progeny virions (lag/eclipse time), the capacity of infected cells to produce virions (yield) and virus/host population densities (multiplicity of infection). However, the optimum can also vary according to mutation rate. Bacteriophage ϕX174 experimental populations treated with the nucleoside analog 5-fluorouracil showed increased mutation frequency and reduced growth [92]. As opposed to other viruses, polymerase fidelity variants cannot evolve in response to this type of treatment because bacteriophage ϕX174, as well as other small DNA viruses, does not code for a polymerase. Interestingly, 5-fluorouracil selected for an amino acid replacement in the N-terminal region of the phage lysis protein (V2A). This change conferred partial resistance to the drug, but also delayed lysis [93]. In turn, delayed lysis was concomitant with an increase in the viral yield per cell, since progeny virions had more time to accumulate intracellularly. Therefore, at the population level, growth of the V2A variant occurred through longer infection cycles with increased per-cell productivity. However, because the virus replicates following a stamping machine model, each infection cycle should involve only one round of copying regardless of lysis time. As a result, population growth required fewer total rounds of copying in the delayed lysis variants than in the wild-type, meaning that mutations had fewer opportunities to accumulate (Fig. 3b). Therefore, delayed lysis increased the ability of the phage to tolerate mutagenesis.

## Template-dependent effects on mutation rate

**Fig. 4:**
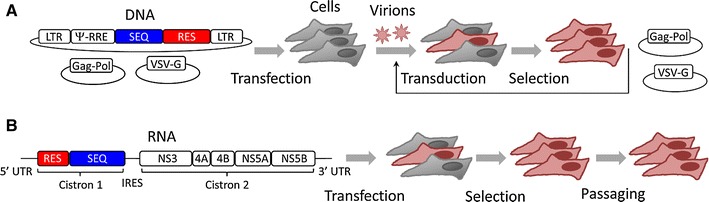
Cell culture systems for the accumulation of mutations in the absence of selection. **a** HIV-1 shuttle vector. The vector contains only cis-acting HIV-1 sequences such as the Rev-responsive element (RRE), the encapsidation signal (Ψ), and the long terminal repeats (LTR). A resistance gene (RES, *red*) is inserted to allow for the selection of cells containing the vector. Any (short) sequence of interest (SEQ, *blue*), including HIV sequences, can be cloned in the shuttle vector and propagated in the absence of selection. The shuttle vector DNA is co-transfected with helper plasmids encoding the Gag (capsid) and Pol (RT, integrase) proteins as well as a viral glycoprotein suited for transducing a given cell line (here vesicular stomatitis G protein, VSV-G, which has a broad tropism). Pseudotyped viruses are produced, used for transduction, and cells carrying the retroviral shuttle vector are selected with the appropriate antibiotic. The infection cycle can be restarted at any time by transfecting the two helper plasmids. The SEQ DNA is then extracted, PCR-amplified, and sequenced to score mutations. **b** HCV replicon. Two cistrons are separated by an internal ribosome entry site (IRES). The *right* cistron encodes HCV non-structural (NS) proteins required for replication, but lacks the envelope proteins and hence does not support viral budding. The *left* replicon carries a resistance gene to select cells carrying the replicon. Reporters such as luciferase can be also cloned in this cistron. Since these play no function, they can be replaced with any short sequence of interest (SEQ), including HCV sequences. Replicon RNA is obtained by in vitro transcription and transfected into Huh7 hepatoma cells. Cells are selected using the appropriate antibiotic and passaged before confluence to allow vigorous replication of the viral RNA. The SEQ RNA is reverse-transcribed, PCR-amplified, and sequenced to score mutations

The fidelity of a given polymerase varies according to certain template properties. It is well known that misalignments at homopolymeric runs can cause frameshift mutations and base substitutions [94]. Sequence context may influence the fidelity of HIV-1 RT by modulating enzyme binding and dissociation [95]. Also, RNA secondary structures have been shown to promote template switching, a process that does not lead to new mutations but produces recombinant viruses [96–98]. In recent work, we found that RNA structure can also modulate the fidelity of HIV-1 RT [99]. Shuttle vectors are systems in which most or all sequences except essential cis-acting elements (such as the Rev-responsive element or long terminal repeats) have been removed from the viral genome. Shuttle vectors allow propagating HIV-1 in the absence of selection because all required functions are provided in trans by helper plasmids that are freshly provided in each infection cycle [100] (Fig. 4a). The shuttle vector simply carries forward sequences of interest, which can be reporter genes for selecting and visualizing transduced cells, or transgenes for engineering purposes. However, the vector also accepts HIV-1 sequences. These will have no role in the infection cycle, as they are not expressed. Because selection is absent, such HIV-1 sequences cloned in a shuttle vector can be used for interrogating the viral mutation rate in cognate templates, which is helpful for testing the effects of sequence context or RNA structure on mutation rate.

Using this system, we recently characterized the distribution of mutations along the HIV-1 envelope, integrase, *vif*, and *vpr* genes [99]. We found that a 1 kb region encompassing the V1–V5 loops of the gp120 envelope protein accumulated approximately three times fewer mutations than other regions of the HIV-1 genome. This coldspot mapped to the outermost domains of gp120, which are preferred targets of circulating antibodies and show extensive glycosylation. Examination of this region revealed two differential properties. First, it contained fewer-than-expected GG and GA dinucleotides, which are the preferred sequence contexts of APOBEC3, as previously discussed [101, 102]. As a result, APOBEC-driven G-to-A mutations were less frequent in V1–V5 than in other genome regions. Second, using the RNA structure morel previously determined by selective 2′-hydroxyl acylation analyzed by primer extension (SHAPE), we found that this 1 kb region exhibited significantly fewer RNA base-pairs than other regions of the envelope gene [103]. To more directly test the effect of RNA structure on HIV-1 RT fidelity, we used in vitro polymerization assays with two different templates: a random sequence and RNA from potato spindle tuber viroid, which shows a marked, stem-like secondary structure [104]. We found an increased RT error rate in the viroid RNA compared to the random sequence, suggesting that RT fidelity decreases in highly structured RNA.

Using a conceptually similar approach, we recently characterized the accumulation of mutations along the HCV genome under weak or no selection using a bicistronic replicon by cloning HCV sequences at a site commonly used for inserting reporter genes (Fig. 4b). This revealed extreme mutation rate variations across individual nucleotide sites of the viral genome, with differences of orders of magnitude even between adjacent sites [105]. In that system, we found little or no effect of RNA structure on mutation rate, but a more significant effect of base identity, such that A and U bases were more prone to mutation than G and C.

## Targeted hyper-mutation in viruses

The finding that HIV-1 has a reduced mutation rate in the genome region encoding the outermost domains of the gp120 envelope protein reveals an uncoupling between mutation rate and genetic diversity, as these domains are the most variable regions of the HIV-1 genome, mainly as a consequence of immune pressure [106]. This indicates that HIV-1 has not evolved the ability to target mutation to regions wherein they are more likely to be needed for adaptation. A possible evolutionary explanation for the gp120 V1–V5 coldspot is that some APOBEC-driven mutations favored by immune pressure during HIV-1 evolutionary history resulted in loss of APOBEC targets, leading to a subsequent reduction in mutation rate. Similarly, strong selection at the protein level may have favored amino acid replacements within this region even at the cost of disrupting pre-existing RNA secondary structures and, as a consequence, these RNA structural changes would have modified replication fidelity [99]. In HCV, we found no significant differences in mutation rate across genes [105], as opposed to genetic variation, which concentrates in specific genomes regions including external domains of the E2 envelope protein [107]. This again supports the view that RNA viruses cannot target mutations to specific genomes regions to improve their adaptability.

This contrasts with bacteria and DNA viruses, in which mechanisms of error-prone replication have evolved at specific *loci* involved in host-pathogen interactions [108–110]. A well-characterized system of mutation targeting, called diversity-generating retro-elements (DGRs), is found in large DNA bacteriophages [110]. DGRs are typically located in genes involved in host attachment, a step of the infection cycle that is subject to rapid changes depending on host species availability. DGRs were first identified in the *Bordetella* BPP-1 bacteriophage [111], and always contain two sequence repeats called variable repeat (VR) and template repeat (TR). The BPP-1 VR is located in the 3′ end of the *mtd* gene (major tropism determinant), which encodes a tail fiber protein. The TR is located downstream of the VR and has a highly conserved sequence, in contrast to the VR. An RT is also encoded by the DGR and synthesizes a cDNA from the TR transcript, a process during which extensive mutagenesis of adenines takes place by a key unknown mechanism. The cDNA is then transferred to the VR, producing a large number of variants of the *mtd* gene capable of interacting with new host ligands [112]. Some hypervariable genes in DNA viruses from the human lower gastrointestinal tract show homology with the BPP-1 DGR, and most of these *loci* are linked to RT genes, suggesting the presence of DGRs [113]. DGRs have also been described in plasmids, bacterial and archaeal chromosomes, and archaeal viruses [114–116]. It therefore appears that at least some prokaryotic DNA viruses have evolved the ability to target mutations to specific regions, as opposed to RNA viruses.

## Interplays between mutation and recombination

Diversity-generating retro-elements have not been described in eukaryotic viruses, but these viruses can use other mechanisms of mutational targeting that involve recombination. The inverted terminal repeats of vaccinia virus contain 10–100 base repeated sequence motifs known to experience frequent unequal crossover events and rapid changes in copy number [117, 118]. Recombination has been shown to promote the rapid production of genetic diversity in other genome regions of the vaccinia virus involved in immune escape and the colonization of novel hosts. Protein kinase R (PKR) is a central effector of innate antiviral immunity that induces translational shutoff, modifies protein phosphorylation status, alters mRNA stability, and induces apoptosis [119]. Poxvirus proteins K3L and E3L block PKR and have evolved as antagonists of innate immune responses in a host-specific manner [120, 121]. Experimental deletion of E3L renders vaccinia virus more susceptible to host antiviral responses, imposing a strong selection pressure in the other PKR suppressor K3L to increase its function [108]. Serial transfers of E3L-deleted vaccinia virus led to an elevated K3L copy number, a recombination-driven process that allowed the virus to overexpress this gene. This gain-of-function mutation had a direct fitness benefit, but also increased the number of available targets for the appearance of subsequent selectively advantageous point mutations in K3L. Remarkably, upon selection of these mutants K3L copy numbers were again reduced. Hence, recombination led to an evolutionary process characterized by expansion and contraction of a specific genome region. These so-called genomic accordions have been posited to mediate adaptive duplications in other poxviruses such as myxoma virus [122].

Interesting interplays between recombination and mutation rates have also been recently found in RNA viruses. These two processes are primarily controlled by the viral polymerase since, in RNA viruses, recombination takes place when the viral polymerase switches between different template genomes present in the same cell [123]. The estimated recombination rates of different riboviruses and retroviruses correlate positively with estimated mutation rates [124]. High mutation rates confer viruses the ability to rapidly produce advantageous mutations, but also inflate the genetic load of the population. In turn, frequent recombination allows beneficial mutations to unlink from deleterious genetic backgrounds, as well different beneficial mutations to be combined into the same genome. As such, recombination is expected to enhance adaptation when a large number of alleles coexist in the same population, a scenario that typically takes place at high mutation rates [125]. Experimental evidence supporting the joint effects of recombination and mutation rates in viral adaptability has been recently obtained using poliovirus polymerase mutants that individually alter replication fidelity or recombination rate [126]. In another recent work, a low-fidelity variant of Sindbis virus was found to exhibit increased recombination [127]. This variant showed low fitness and a greater tendency to accumulate defective interfering particles (i.e. mutant viruses with large deletions that depend on and interfere with the wild-type infection cycle). Therefore, it appears that high mutation and recombination rates enhance viral adaptability, but only up to a certain point, beyond which both processes contribute to the accumulation of deleterious alleles in the population.

## Conclusions

Viral mutation rates are determined by multiple processes, including polymerase intrinsic fidelity, replication mode, 3′ exonuclease activity, spontaneous nucleic acid damage, access to post-replicative repair, editing by host-encoded deaminases, imbalances in nucleotide pools, template sequence context, and template structure, as summarized in Table 2. Some of these processes underlie large-scale patterns of variation among viruses, such as differences between RNA and DNA viruses, between viruses with small and large genomes, and between single-strand and double-strand viruses, but important mechanistic aspects behind these differences still remain uncharacterized. Furthermore, mutation rates are not static and can evolve in response to selective pressures, as exemplified by fidelity variants selected under mutagenic conditions in a variety of viruses. In addition to polymerase fidelity, other mutation rate-determinants such as access to DNA repair may have also changed in response to selective pressures during viral evolution.

**Table 2:** Molecular determinants of viral mutation rates

	Higher mutation rate	Lower mutation rate
Global genomic features	RNA, single-strand, short	DNA, double-strand, large
Specific template features	RNA base-pairs, sequence repeats	–
Replication mode	Semi-conservative	Stamping machine
Fidelity mechanisms	–	3′ Exonuclease, repair
Host-encoded proteins	APOBEC, ADAR	UNG, MutHLS/Dam
Other host factors	Unbalanced dNTP pools, ROS	–

In RNA viruses, both low- and high-fidelity polymerase variants tend to have a negative impact in viral fitness in complex environments, suggesting that RNA virus mutation rates have been evolutionarily optimized. Given that DNA virus mutation rates are substantially lower than those of RNA viruses this also suggests that DNA viruses show suboptimal mutation rates for adaptation to rapidly changing environments, despite RNA and DNA viruses sharing similar lifestyles. It appears that large DNA viruses have adopted a different and more elaborate strategy consisting of targeting mutations to specific genome regions subject to rapidly varying selective pressures, such as genes encoding attachment proteins or inhibitors of innate immunity responses. Mutation targeting mechanisms such as DGRs and recombination-driven gene copy amplification are probably not accessible to small DNA viruses with compact genomes. Furthermore, mutation rate evolution in small DNA viruses is further constrained by the fact they do not encode autonomous replication systems. Therefore, small DNA viruses should rely on repair avoidance and on use of host-encoded error-prone DNA polymerases to elevate their mutation rates and achieve faster adaptation. Elucidating the mutational mechanisms of small DNA viruses is a current challenge in virus molecular biology and evolution. Other exciting unresolved questions include unveiling the interplays between mutation and recombination, the roles played by viral accessory proteins in determining mutation rates, the effects of host-encoding enzymes on viral diversity and evolution, whether mutation accumulation can be evolutionary adjusted by modifying viral replication modes, and how template sequences regulate viral mutation rates.

## Abbreviations

APOBEC: Apolipoprotein B mRNA-editing catalytic polypeptide-like enzymes
ADAR: dsRNA-dependent adenosine deaminase
AZT: Azidothymidine
CTL: Cytotoxic T lymphocyte
DDR: DNA damage response
HBV: Hepatitis B virus
HCV: Hepatitis C virus
MMR: Methyl-directed mismatch repair
PKR: Protein kinase R
ROS: Reactive oxygen species
SHAPE: Selective 2′-hydroxyl acylation analyzed by primer extension
UNG: Uracil DNA glycosylases
